# Characteristics and Treatment Outcomes of Patients with Drug-Resistant Tuberculosis in Selected Facilities of O.R. Tambo District Municipality, Eastern Cape, South Africa: A Retrospective Cohort Study

**DOI:** 10.3390/epidemiologia7050137

**Published:** 2026-09-30

**Authors:** Yanga Somjobo, Lindiwe Modest Faye, Mojisola Clara Hosu

**Affiliations:** Tuberculosis and Associated Research and Innovation Platform (TARIP), School of Pathology, Faculty of Medicine and Health Sciences, IYunivesithi Walter Sisulu, Mthatha 5100, South Africa; yangasomjobo@gmail.com (Y.S.); lfaye@wsu.ac.za (L.M.F.)

**Keywords:** drug-resistant tuberculosis, multidrug-resistant tuberculosis, epidemiology, tuberculosis burden, TB/HIV coinfection, O.R. Tambo District Municipality, Eastern Cape

## Abstract

**Background**: Tuberculosis (TB) remains a major global health concern, particularly in low- and middle-income countries, due to its high prevalence, mortality, and economic impact. The emergence of drug-resistant tuberculosis (DR-TB) has intensified this burden, posing significant challenges to disease control and management. Understanding the epidemiology of DR-TB is crucial for improving prevention strategies, guiding treatment interventions, and informing public health policies aimed at reducing its spread and impact. **Methods:** A retrospective cohort study was conducted using medical records of patients diagnosed with DR-TB between January 2020 and December 2024 at decentralized DR-TB clinics in the O.R. Tambo District Municipality, Eastern Cape, South Africa. Treatment outcomes were collected from available records through August 2025. Data on demographic, clinical, and treatment outcome variables were collected using a structured abstraction tool. Data was analyzed using SPSS version 30 (IBM Corp., Armonk, NY, USA). **Results**: A total of 385 participants were included in the study, with a mean age of 38.6 years. Males accounted for 62.3% of cases. Most participants (94.3%) resided in rural areas, and 62% were HIV-coinfected. Rifampicin-resistant TB was the most common type (51%), followed by MDR-TB (38%). A significant association was observed between age and HIV status (*p* = 0.001). Treatment outcomes showed that 40.8% of patients were cured, while 11.7% died, with mortality highest among older and HIV-positive patients. Comorbidities reported included hypertension and diabetes mellitus. **Conclusions**: Most patients included in this facility-based cohort were males, aged 25–39 years, rural residents, living with HIV, with suboptimal treatment outcomes and a substantial comorbidity burden. Strengthened integrated TB/HIV care, early diagnosis, and targeted interventions are needed to improve DR-TB outcomes in this setting.

## 1. Introduction

Tuberculosis (TB) is a contagious disease caused by *Mycobacterium tuberculosis*, primarily affecting the lungs, and is characterized by a prolonged incubation period and easy transmission. It remains a significant global public health challenge despite improvements in treatment and prevention [1,2]. According to the 2025 WHO global report, there were approximately 10.7 million new TB cases globally, with 54% men, 35% women, and 11% children. In the same year, an estimated 1.23 million deaths were recorded, with a case fatality rate of 11.5% [3]. TB continues to disproportionately affect low- and middle-income countries (LMICs), particularly in sub-Saharan Africa, where poverty, overcrowding, malnutrition, HIV co-infection, and limited healthcare access perpetuate disease transmission and poor treatment outcomes [4,5].

Drug-resistant tuberculosis (DR-TB) has emerged as a major public health challenge, threatening progress toward the global End TB Strategy targets [6]. DR-TB occurs when *Mycobacterium tuberculosis* develops resistance to one or more first-line anti-TB drugs, primarily due to inadequate treatment adherence, poor-quality medications, delayed diagnosis, interrupted drug supply, and ongoing transmission of resistant strains within communities [6,7,8]. The most concerning forms include rifampicin-resistant TB (RR-TB), multidrug-resistant TB (MDR-TB), pre-extensively drug-resistant TB (pre-XDR-TB), and extensively drug-resistant TB (XDR-TB). In 2024, approximately 0.40 million cases were MDR/RR-TB, with an estimated 0.15 million deaths, underscoring the persistent challenge of DR-TB in global TB control efforts [3]. Although the global burden of MDR/RR-TB has gradually declined since 2015, DR-TB remains a significant challenge in resource-limited settings because of prolonged treatment regimens, adverse drug reactions, low treatment success rates, and increased healthcare costs. The economic burden of DR-TB is substantial, with more than 81% of affected households facing catastrophic healthcare expenses for treatment [9].

The African region continues to bear a disproportionately high burden of TB. According to the WHO African Regional Office, about 57,000 people in Africa developed MDR/RR-TB in 2024, accounting for nearly 15% of global DR-TB cases [10]. That year, roughly 390,000 TB cases in the region were linked to HIV infection, representing about one-third of all TB cases there [10]. The concurrent spread of TB and HIV poses significant challenges for disease control, especially in countries like South Africa, where HIV-induced immunosuppression heightens vulnerability to TB infection and progression to active disease [11,12].

South Africa remains among the 30 countries with the highest TB burden globally and faces significant challenges related to TB/HIV co-infection and DR-TB. According to the 2025 WHO Global TB Report, despite substantial investments in prevention and treatment, South Africa still has one of the world’s highest TB incidence rates [3]. The country has made progress by expanding rapid molecular diagnostics, such as GeneXpert MTB/RIF, and adopting shorter, all-oral DR-TB treatment regimens [13]. Nonetheless, DR-TB continues to impede TB control efforts because of persistent transmission, delayed case detection, socioeconomic disparities, and challenges with treatment adherence.

National surveillance data from the South African District Health Information System (DHIS) indicate that the Eastern Cape Province remains among those with persistently high TB notification rates and a substantial DR-TB burden [14]. The province is marked by widespread poverty, unemployment, inadequate housing, and limited access to healthcare services, all of which contribute to ongoing TB transmission [15,16,17]. Within the Eastern Cape, the O.R. Tambo District Municipality has consistently been identified as a high-burden district for TB and DR-TB. The district experiences considerable socioeconomic deprivation, high HIV prevalence, and healthcare resource constraints, which together increase vulnerability to TB infection and lead to poor treatment outcomes.

Despite ongoing TB control efforts at the national and provincial levels, detailed district-level data on the distribution, trends, and burden of DR-TB remain scarce in many rural and underserved areas of South Africa. Such localized epidemiological information is vital for guiding targeted public health actions, strengthening surveillance, optimizing resource allocation, and improving treatment outcomes in affected communities [1,18]. Additionally, identifying demographic and clinical factors associated with DR-TB at the district level is crucial for developing strategies to reduce transmission and achieve the country’s TB elimination goals.

## 2. Materials and Methods

### 2.1. Study Design

This retrospective cohort study was conducted in two sub-districts of the O.R. Tambo municipality in the Eastern Cape, namely King Sabata Dalindyebo and Ingquza Hill. Records of patients diagnosed with DR-TB between January 2020 and December 2024 were reviewed. Treatment outcomes were tracked and extracted from available medical records through August 2025 to ensure patients diagnosed toward the end of the study period had sufficient time to reach a documented treatment outcome.

### 2.2. Study Setting

The Eastern Cape Province ranks fourth among South Africa’s nine provinces, with a population of 6.67 million, representing 11.1% of the country’s total population. The Eastern Cape’s share of national GDP was 7.6% [15]. The O.R. Tambo District covers approximately 12,095.1 km^2^. The district is designated a Category C2 Municipality, indicating it is predominantly rural. The study was conducted at two selected healthcare facilities in the O.R. Tambo District Municipality in the Eastern Cape Province. One facility is a regional hospital, and the other is a primary healthcare clinic (PHC). Both facilities serve as DR-TB treatment initiation and follow-up centers within the decentralized DR-TB care model in the Eastern Cape Province. The two facilities were purposively selected because they provide decentralized DR-TB diagnostic, treatment initiation, monitoring, and follow-up services and collectively manage a substantial proportion of patients with DR-TB within the district. Including facilities at both the regional hospital and PHC levels also enabled the study to capture patients receiving DR-TB care at different levels of the decentralized healthcare system.

### 2.3. Study Population

The study included patients diagnosed with DR-TB between January 2020 and December 2024 who received care at two healthcare facilities designated as decentralized DR-TB treatment sites in two sub-districts of the O.R. Tambo District Municipality. All available patient records meeting the eligibility criteria during the study period were reviewed; no sampling of eligible records was performed. Patients of all ages, including pediatric patients, were eligible for inclusion. Participants were included if they had a microbiologically confirmed diagnosis of DR-TB during the study period, resided within the O.R. Tambo District Municipality, and received treatment and/or follow-up care at either of the participating decentralized DR-TB facilities. To prevent duplication, each patient was included only once in the analytical dataset. Records were cross-checked using available unique patient identifiers and relevant demographic and clinical information, including patient folder/registration numbers, age/date of birth, sex, facility records, and treatment initiation information, as available in the source records. Where multiple records or treatment entries referred to the same patient and the same DR-TB treatment episode, they were consolidated into a single patient record. When duplicate entries were identified across the study records, only one complete record corresponding to the eligible treatment episode was retained for analysis. Thus, the unit of analysis was the individual patient rather than individual clinical visits or repeated entries. For patients with more than one distinct DR-TB treatment episode during the study period, only the first eligible episode was included.

Patients were excluded if DR-TB was not microbiologically confirmed, if they resided outside the O.R. Tambo District Municipality, if their records were duplicates of an already included treatment episode, or if the available clinical record contained insufficient information to establish eligibility for the study.

### 2.4. Data Collection

Data was extracted from patient files using a structured data abstraction tool. Collected variables included demographics, clinical information, comorbidities, treatment start and end dates, and treatment outcomes. Although the diagnosis period extended to December 2024, treatment outcome information was collected from available records through August 2025 to allow sufficient follow-up for patients diagnosed near the end of the study period. Data was captured in Excel, cleaned, and exported to SPSS for statistical analysis.

### 2.5. Data Analysis

Data were entered into Microsoft Excel and analyzed using SPSS version 30 (IBM Corp., Armonk, NY, USA). Descriptive statistics were used to summarize participants’ demographic and clinical characteristics. Categorical variables were presented as frequencies and percentages. Continuous variables were summarized using means and standard deviations (SD), as well as medians and interquartile range (IQR) or the 25th and 75th percentiles, as appropriate. Pearson’s chi-square (χ^2^) test of independence was used to assess associations between categorical variables, including gender, age, residence, and HIV status. Fisher’s exact test was applied where the assumptions of the chi-square test were not met, particularly when expected cell frequencies were <5. All statistical tests were two-sided, and statistical significance was set at *p* < 0.05. Missing data resulted from information not recorded in the available medical records and were handled using a variable-specific complete-case approach. Participants with missing or undocumented information for a particular variable were excluded only from analyses involving that variable, while remaining eligible for analyses of other variables. Consequently, the denominator for each analysis reflected the number of participants with available data for the variables under consideration, and percentages were calculated using the corresponding available-case denominator.

### 2.6. DR-TB Classification

Drug resistance was determined through routine diagnostic testing performed by the National Health Laboratory Service (NHLS) in accordance with the South African National Tuberculosis management guidelines. Rifampicin resistance was initially detected by Xpert MTB/RIF Ultra, and first- and second-line drug resistance was further characterized using GenoType MTBDRplus version 2.0 and MTBDRsl version 2.0 line probe assays and/or phenotypic drug susceptibility testing (DST). Patients were classified according to the South African National Department of Health Guidelines for the Management of DR-TB and the WHO revised definitions.

### 2.7. Operational Definitions

RR-TB is defined as rifampicin-resistant TB without confirmed isoniazid resistance. MDR-TB is defined as TB caused by *Mycobacterium tuberculosis* resistant to at least both rifampicin and isoniazid. Pre-XDR-TB is defined as MDR/RR-TB with additional resistance to any fluoroquinolone. XDR-TB is defined as MDR/RR-TB with resistance to any fluoroquinolone and at least one additional WHO Group A drug (bedaquiline or linezolid).

## 3. Results

### 3.1. Demographic Characteristics of Study Population

A total of 385 participants were included in the analysis. Over 60% of the population were males, with almost 60% in the 18–39 age bracket. The mean age of participants was 38.6 years (SD = ±14.2), with a median age of 37 years. The age distribution ranged from 25 years at the 25th percentile to 48 years at the 75th percentile. Of the 383 participants with documented HIV status, 238 (62.1%) were HIV-positive, and 145 (37.9%) were HIV-negative, while HIV status was unavailable for two participants. The study population was predominantly rural, with 363 (94.3%) participants residing in rural areas, compared with 22 (5.7%) from peri-urban areas. HIV positivity was particularly concentrated among participants aged 25–39 years (52.1%) and 40–53 years (31.1%), and age group was significantly associated with HIV status (*p* < 0.001). Among participants with known HIV status, males accounted for 59.7% of HIV-positive participants compared with 66.2% of HIV-negative participants. Females represented 40.3% and 33.8% of HIV-positive and HIV-negative participants, respectively. The difference in HIV status according to gender was not statistically significant.

Participants aged 25–39 years constituted the largest age group (46.2%) (Figure 1). Age distribution differed substantially according to HIV status. More than half of HIV-positive participants were aged 25–39 years (52.1%), compared with 36.6% of HIV-negative participants. Similarly, 31.1% of HIV-positive participants were aged 40–53 years, compared with 17.9% of HIV-negative participants. In contrast, participants aged ≤24 years were considerably more common among the HIV-negative group (24.8%) than among the HIV-positive group (5.5%). Those aged ≥54 years were also more frequent among HIV-negative participants (20.7%) than HIV-positive participants (11.3%). This association between age group and HIV status was statistically significant (*p* < 0.001).

### 3.2. Patterns of Drug-Resistant TB

By DR-TB type, RR-TB accounted for 51% of cases, followed by MDR-TB (38%), Pre-XDR-TB (5%), and XDR-TB (0.8%) (Table 1). Across all resistance types, the 25–39-year age group remained the most affected. RR-TB and MDR-TB cases peaked in this group (47.5% and 46.9%, respectively). Pre-XDR-TB was more frequent among older adults aged 40–53 years (40%), whereas all XDR-TB cases occurred in individuals aged 25–39 years. HIV status was documented for 383 (99.5%) of 385 participants. Of those with available HIV-status information, 238 (62.1%) were HIV-positive and 145 (37.9%) were HIV-negative. HIV status was not documented for 2 patients (0.5%). Differences in the distribution of HIV co-infection were observed across DR-TB resistance categories.

### 3.3. Treatment Outcomes

Of the 385 participants, 157 (40.8%) were cured and 91 (23.6%) completed treatment. Thirty-five (9.1%) participants were lost to follow-up, 11 (2.9%) experienced treatment failure, and 45 (11.7%) died. A further 38 (9.9%) were transferred out, while the outcomes of eight participants are not accounted for (Table 2). The extended follow-up period enabled the capture of treatment outcomes for patients diagnosed toward the end of 2024, including those receiving longer treatment regimens. Treatment regimens and DR-TB characteristics of the study population, stratified by treatment outcomes, are shown in Table 3.

## 4. Discussion

The present study found that most patients in this facility-based cohort were male and in the young, economically active age group. The mean age of participants was 38.6 years, with 59% aged 18–39 years. This demographic pattern aligns with findings from South Africa and other high-burden countries, where DR-TB predominantly affects young and middle-aged adults. For example, a recent DR-TB hotspot study in the O.R. Tambo District reported a mean age of 37.5 years, with most patients in the 20–59-year range [19]. Similar observations have been reported in Ethiopia, Nigeria, and Zimbabwe, where DR-TB disproportionately affects socially and economically active adults [20,21,22]. In Ethiopia, Bonsa et al. [23] found that nearly half of MDR-TB patients were 25–44 years of age, a pattern attributed to greater social interaction, mobility, and the high prevalence of HIV among younger adults. Comparable findings from India and Central Asia have also shown a higher burden of MDR/RR-TB among middle-aged individuals, further indicating that DR-TB primarily affects economically productive populations [24,25]. Collectively, these findings suggest ongoing community transmission among mobile, working-age adults and highlight the substantial consequences of DR-TB, including household transmission, loss of productivity, and increased strain on health systems.

In this study, males accounted for 62.3% of cases, consistent with other South African and international research. The WHO reports consistently indicate that TB is more common in men than in women globally [3,26]. The predominance of males is epidemiologically important, as men frequently constitute a greater proportion of notified TB cases. However, the absence of a statistically significant association between sex and HIV status suggests that, within this DR-TB population, the observed male predominance was not dependent on HIV infection. Male dominance in DR-TB cases has been repeatedly observed in cohorts from South Africa, Nigeria, and Ethiopia [19,20,27]. Consistent with our study findings, Faye et al. [19] observed that 56.1% of participants were male, whereas Ogunsina et al. [28] reported that 67.6% were male and 32.4% were female. Gender differences in TB are linked to occupational exposure and behavioral factors such as delayed healthcare seeking, smoking, alcohol use, and poor treatment adherence among men [29,30,31]. Men’s occupational mobility increases the risk of treatment interruption and the development of DR-TB, while family and community support promote treatment adherence and reduce this risk [32]. Active, young working men, often away from family and community, face a higher risk of DR-TB. These patterns are more prominent in men than in women [29]. In Asia, male migration primarily drives disease spread, whereas in sub-Saharan Africa, a high female HIV burden due to structural vulnerabilities influences disparities. These regional differences highlight how socioeconomic and demographic factors shape gender differences in public health [33,34,35,36].

The predominance of rural residents in the cohort should be viewed as a characteristic of the population served rather than as evidence that rural residence independently predicts poor treatment outcomes. Nevertheless, because most patients receiving DR-TB care in this study live in rural communities, the organization of district services should account for the geographical context in which care is delivered. Broader strategies, such as decentralized DR-TB care, strengthened referral systems, and community-based treatment support, are consistent with South African TB program policy and published evidence, but their effectiveness was not directly evaluated in this study. Rural populations may face challenges related to geographic distance, transportation, continuity of care, and access to specialized health services. Nevertheless, because rural residence was common in both HIV-positive and HIV-negative groups and the difference was not statistically significant, the present data do not demonstrate an association between residence and HIV status. Despite decentralization efforts, DR-TB service delivery in the O.R Tambo District remains constrained by geographic and socioeconomic barriers, leading to poor adherence and treatment interruptions [37]. Globally, an alarming health disparity persists between urban and rural populations [38]. Rurality has been linked to delayed diagnosis, limited treatment access, transportation barriers, and interrupted continuity of care in several African countries [17,39]. Studies from rural KwaZulu-Natal and Mozambique report that patients frequently experience diagnostic delays and poorer treatment outcomes due to limited healthcare infrastructure and inadequate access to specialized DR-TB services [40,41,42]. These findings underscore the urgent need to strengthen rural health systems and expand access to timely DR-TB diagnosis and treatment to reduce the rural burden of the disease.

The high prevalence of HIV co-infection observed among patients with DR-TB demonstrates the continued intersection of the TB and HIV epidemics in this setting. The significant association between age and HIV status further highlights the influence of HIV on the epidemiology of DR-TB, particularly among younger and middle-aged adults. Similar HIV co-infection rates have been reported in KwaZulu-Natal and the Eastern Cape, where HIV prevalence among DR-TB patients often exceeds 50% [43,44]. Likewise, studies from Lesotho and Eswatini have documented exceptionally high HIV prevalence among MDR/RR-TB patients [45,46]. HIV is a well-established risk factor for adverse DR-TB treatment outcomes, even among patients receiving antiretroviral therapy (ART) [47,48]. HIV-associated immunosuppression increases the risk of severe disease, opportunistic infections, treatment complications, and mortality. In addition, the concurrent management of HIV and DR-TB is complicated by drug-drug interactions, overlapping toxicities, high pill burden, and adherence challenges. These findings underscore the importance of integrated TB-HIV services, adherence support, close clinical monitoring, and multidisciplinary care to improve treatment outcomes, particularly in predominantly rural settings such as the Eastern Cape [49,50]. The observed relationship between HIV co-infection and DR-TB resistance patterns should be interpreted cautiously. Although differences in HIV prevalence were observed across resistance categories, the retrospective cross-sectional design does not allow determination of whether HIV infection preceded the development of drug resistance or contributed to its increase. HIV-associated immunosuppression may increase susceptibility to active TB and complicate treatment [51,52]; however, drug resistance may also reflect prior anti-TB treatment exposure, inadequate or interrupted treatment, healthcare-related factors, or direct transmission of resistant strains. Therefore, the present findings demonstrate an epidemiological association and should not be interpreted as evidence that HIV co-infection causes progression to more extensive drug resistance.

Our study found that RR-TB is the predominant form of drug resistance. This pattern closely mirrors findings from retrospective cross-sectional studies in Limpopo [53,54]. Previous studies have shown that RR-TB is a reliable surrogate marker for MDR-TB because rifampicin resistance is often accompanied by isoniazid resistance [55]. The predominance of RR-TB and MDR-TB in this study should be interpreted within the broader epidemiological context of DR-TB in South Africa, a country with a high burden of both TB and DR-TB. In the O.R. Tambo District, previous studies have reported a substantial burden of RR-TB and MDR-TB, including geographic clustering of cases in socioeconomically disadvantaged communities, suggesting that ongoing transmission contributes to local resistance patterns [19]. In this high-burden setting, three distinct mechanisms may explain the elevated rates of dual resistance to rifampicin and isoniazid: community transmission of resistant clones, selective pressure from prior treatment exposure, and the concentration of confirmed DR-TB cases due to decentralized referral patterns [56]; however, these mechanisms were not directly assessed in the present study. The clinical and referral context of the present study is also relevant. Patient records were obtained from a regional hospital and a primary healthcare clinic functioning as decentralized DR-TB treatment facilities; consequently, the study population comprised patients with microbiologically confirmed DR-TB rather than an unselected population of patients with TB. This referral and case-selection process would be expected to enrich the cohort for RR- and MDR-TB cases and may partly explain the high proportions observed. Furthermore, the high proportion of RR-TB may also reflect South Africa’s extensive use of rapid molecular diagnostics, such as Xpert MTB/RIF and Xpert MTB/RIF Ultra, which facilitate early detection of rifampicin resistance and referral of patients with RR-TB to DR-TB treatment programs. Xpert MTB/RIF has high diagnostic accuracy in detecting rifampicin resistance and has been widely implemented in South Africa’s laboratory system. Because rifampicin resistance is frequently accompanied by isoniazid resistance, substantial overlap between RR-TB and MDR-TB is expected in high-burden settings [57,58]. In this study, however, RR-TB and MDR-TB were analyzed as mutually exclusive categories, with MDR-TB defined as resistance to both rifampicin and isoniazid.

Previous anti-TB treatment may also contribute to the observed resistance patterns, as poor adherence, treatment interruptions, inadequate drug exposure, and inappropriate treatment regimens can promote the development of drug resistance [3,58]. However, primary transmission of resistant strains is increasingly recognized as a major driver of the DR-TB epidemic in South Africa. Therefore, the observed resistance patterns likely reflect a combination of acquired resistance and community transmission of resistant strains. Furthermore, the study population was drawn from healthcare facilities managing patients with DR-TB rather than from all TB patients in the district. As a result, the high proportions of RR-TB and MDR-TB reflect the resistance profile of patients accessing DR-TB care and should not be interpreted as the prevalence of drug resistance among all TB patients in O.R. Tambo District.

The predominantly rural composition of the cohort may have influenced the observed resistance patterns. Rural communities often face geographic barriers to healthcare access, diagnostic delays, socioeconomic challenges, and difficulties maintaining continuity of care, all of which may contribute to ongoing transmission of drug-resistant strains and poor treatment outcomes. Consistent with this, a previous study in O.R. Tambo District identified social and structural factors as important determinants of the local DR-TB epidemic [19]. Taken together, the predominance of RR-TB and MDR-TB in this cohort likely reflects a combination of the high underlying burden of DR-TB in the district, ongoing transmission of resistant strains, previous anti-TB treatment exposure, referral patterns to DR-TB services, and enhanced detection through molecular diagnostic testing. These findings highlight the need for comprehensive drug-susceptibility testing, timely identification of additional resistance, and strengthened surveillance and control measures for DR-TB in high-burden rural communities.

The treatment outcomes in this study raise concerns. The observed cure rate is notably lower than the figures reported in the WHO global 2025 report and in another South African study [3,53]. These disparities may be due to factors such as the rural setting of O.R. Tambo, high HIV co-infection rates, socioeconomic challenges, and potential gaps in treatment support. The mortality rate, especially among HIV-positive individuals, aligns with data from high HIV-burden areas in sub-Saharan Africa [53,59]. HIV-positive patients face a heightened risk of death due to drug interactions, delayed ART initiation, immunosuppression, opportunistic infections, and treatment complications [60].

The presence of hypertension and diabetes as comorbidities is clinically significant, as these non-communicable diseases (NCDs) may complicate DR-TB treatment, increase medication burden, and contribute to poorer treatment outcomes. Although hypertension and diabetes are not directly caused by TB-HIV coinfection, their coexistence adds to the clinical complexity of managing patients with DR-TB and HIV, often requiring coordinated and multidisciplinary care. Similar findings have been reported in KwaZulu-Natal and among public primary healthcare patients in South Africa, where hypertension, diabetes mellitus, cardiovascular disease (CVD), and other NCDs were associated with increased treatment complexity, poorer TB and DR-TB outcomes, and higher mortality [61,62,63]. These findings highlight the growing dual burden of infectious diseases and NCDs in the Eastern Cape and underscore the need for integrated screening and management at the primary healthcare level, particularly among patients initiating TB treatment.

### 4.1. Implications for Public Health Practice

The findings highlight several priorities for DR-TB control in the O.R. Tambo District Municipality. The predominance of RR-TB and MDR-TB supports continued access to rapid molecular diagnosis and drug-susceptibility testing to facilitate early identification of additional resistance and appropriate treatment. The high burden of HIV co-infection reinforces the importance of integrated DR-TB/HIV services and coordinated treatment monitoring. Furthermore, because the study population was overwhelmingly rural, district DR-TB services should be organized around the realities of geographically dispersed communities, including decentralized treatment and follow-up, effective referral systems, and community-based support. The observed loss to follow-up and mortality further emphasize the need to strengthen treatment monitoring, patient tracing, continuity of care, and documentation of final outcomes. Screening and management of common comorbidities should also be part of comprehensive DR-TB care. Finally, district TB programs should strengthen routine surveillance so that local trends in mortality, cure, and resistance patterns can guide timely decisions on staffing, outreach, and drug supply. These priorities align with South Africa’s current RR-TB clinical guidance and TB recovery strategy.

### 4.2. Limitations of the Study

This study has several limitations. First, the retrospective cohort design relied on routinely recorded data, which may have been missing or incomplete. The cohort nature of the study precluded assessment of temporal or causal relationships between HIV infection and the development or extent of anti-TB drug resistance. Second, the study could describe patterns and associations but could not establish cause-and-effect relationships. Third, the analysis was limited to variables available in patient records, so important factors such as the timing of ART initiation, adherence, socioeconomic hardship, distance to clinic, and resistance to second-line drugs may not have been fully captured. Fourth, the findings come from one district and may not be directly generalizable to all settings in South Africa. Finally, treatment outcome categories were summarized at a single point in time, so longer-term relapse or post-treatment survival could not be assessed.

## 5. Conclusions

This study demonstrates a substantial burden of DR-TB among patients receiving care in the O.R. Tambo District Municipality, with RR-TB and MDR-TB representing the predominant resistance categories. The cohort was predominantly composed of adults of economically productive age, males, rural residents, and people living with HIV. The occurrence of loss to follow-up, treatment failure, and mortality indicates ongoing challenges in DR-TB management and supports strengthening surveillance, treatment monitoring, continuity of care, and documentation of treatment outcomes within the district. The high burden of HIV co-infection further underscores the relevance of integrated DR-TB/HIV care. However, the present analysis did not establish male sex, older age, or rural residence as independent predictors of unsuccessful treatment outcomes; therefore, targeted interventions for these specific groups cannot be recommended on the basis of this study alone. Further longitudinal studies incorporating multivariable analyses are needed to identify independent predictors of unsuccessful DR-TB treatment outcomes and to inform appropriately targeted interventions.

## Figures and Tables

**Figure 1:**
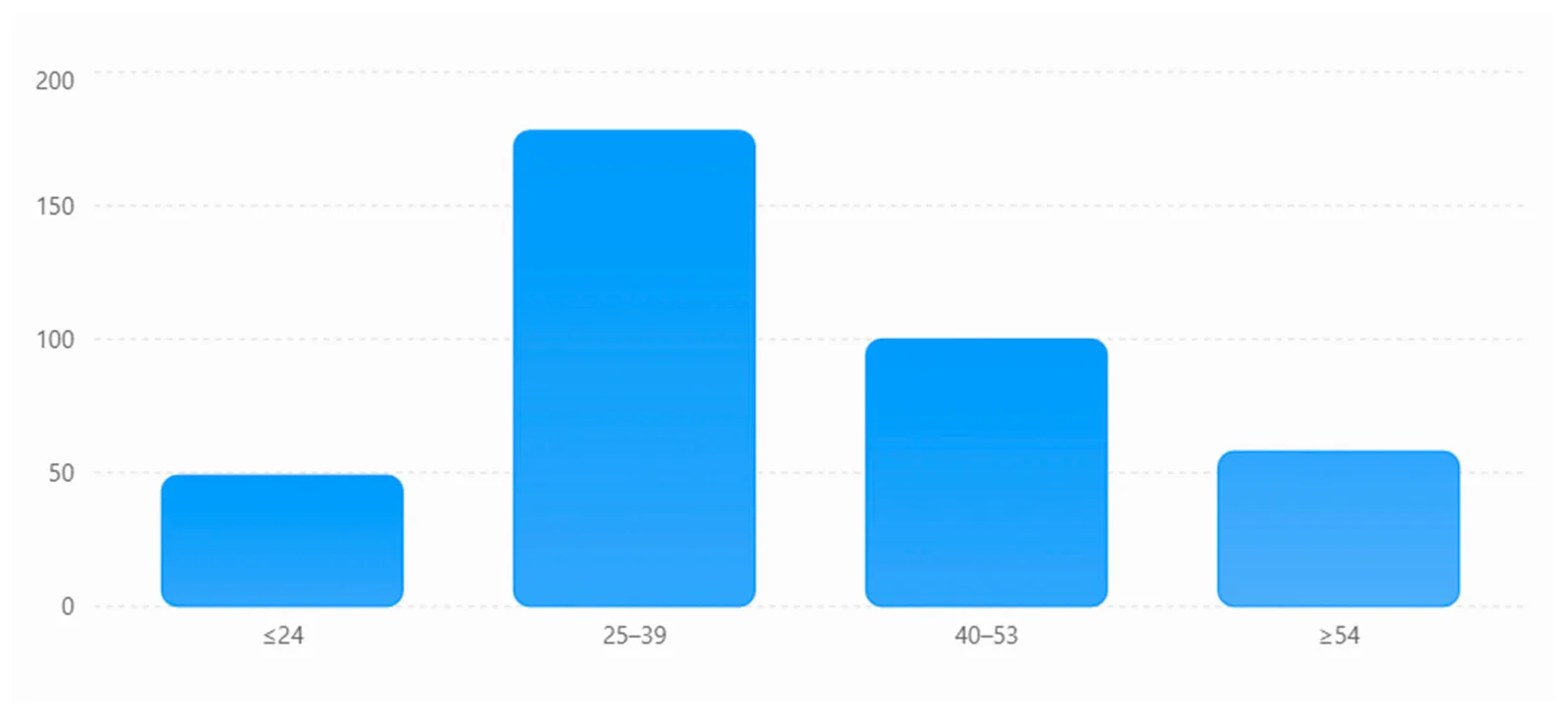
Age distribution of the study population.

**Table 1:** Distribution of DR-TB and HIV coinfection in the study population.

Variables	All (N = 385), n (%)	RR (N = 196), n (%)	MDR (N = 145), n (%)	Pre-XDR (N = 20), n (%)	XDR (N = 3), n (%)
Age					
≤24	49 (12.7)	24 (12.2)	21 (14.5)	1 (5.0)	0 (0)
25–39	178 (46.2)	93 (47.5)	68 (46.9)	7 (35.0)	3 (100)
40–53	100 (26.0)	50 (25.5)	34 (23.5)	8 (40.0)	0 (0)
54+	58 (15.1)	29 (14.8)	22 (15.2)	4 (20.0)	0 (0)
Gender					
Male	240 (62.3)	127 (64.8)	87 (60.0)	10 (50)	1 (33.3)
Female	145 (37.7)	69 (35.2)	58 (40.0)	10 (50)	2 (66.7)
HIV status (n = 383)					
HIV positive	238 (62.1)	120 (61.2)	90 (62.1)	13 (65.0)	3 (100)
HIV negative	145 (37.9)	76 (38.8)	54 (37.2)	7 (35.0)	0 (0)

RR-TB, rifampicin-resistant tuberculosis; MDR-TB, multidrug-resistant tuberculosis; pre-XDR-TB, pre-extensively drug-resistant tuberculosis; XDR-TB, extensively drug-resistant tuberculosis; HIV status was available for 383 of the 385 participants; two participants had undocumented HIV-status information. Percentages for HIV-positive and HIV-negative categories were calculated using participants with available HIV-status data (n = 383).

**Table 2:** Treatment outcomes of the study population.

Treatment Outcome	n	% of Total Cohort (N = 385)
Cured	157	40.8
Treatment completed	91	23.6
Lost to follow-up (LTFU)	35	9.1
Treatment failed	11	2.9
Died	45	11.7
Transferred out	38	9.9
Outcome not accounted for	8	2.1
Total	385	100.0

**Table 3:** Treatment regimen and DR-TB classification of the study population stratified by treatment outcomes.

	Cured n (%)	Treatment Completed n (%)	LTFU n (%)	Failed n (%)	Died n (%)	Transferred Out n (%)
Treatment regimen						
Long	16 (10.2)	14 (15.4)	5 (14.3)	0 (0.0)	9 (20.0)	11 (29.0)
Short	141 (89.8)	77 (84.6)	30 (85.7)	11 (100.0)	36 (80.0)	27 (71.1)
DR-TB classification						
RR-TB	74 (47.1)	50 (55.0)	21 (60.0)	0 (0.0)	29 (64.4)	18 (47.4)
MDR-TB	61 (38.9)	37 (40.7)	10 (28.6)	9 (81.8)	12 (26.7)	13 (34.2)
Pre-XDR-TB	5 (3.2)	3 (3.3)	2 (5.6)	2 (18.2)	2 (4.4)	6 (15.8)
XDR-TB	0 (0.0)	0 (0.0)	2 (5.7)	0 (0.0)	0 (0.0)	1 (2.6)

DR-TB, drug-resistant tuberculosis; RR-TB, rifampicin-resistant tuberculosis; MDR-TB, multidrug-resistant tuberculosis; pre-XDR-TB, pre-extensively drug-resistant tuberculosis; XDR-TB, extensively drug-resistant tuberculosis; LTFU, loss to follow-up. Denominators vary across variables because some information was not documented in the medical records. Percentages were calculated using the number of participants with available data for the relevant variable.

## Data Availability

The aggregated data supporting the findings of this study are presented in this article. The underlying patient-level dataset is not publicly available because it was obtained retrospectively from confidential medical records and contains sensitive clinical information. The ethical and health-authority approvals granted access to these records for the study and did not permit unrestricted public sharing of individual-level data. A deidentified patient-level dataset is also not publicly available because combinations of demographic, clinical, HIV-related, drug-resistance, treatment, facility, and outcome variables may retain a residual risk of participant re-identification, particularly within small or rare clinical subgroups. Public sharing could therefore compromise patient confidentiality and would extend beyond the conditions of the original ethical and institutional approvals.

## Abbreviations

The following abbreviations are used in this manuscript:

ART	Antiretroviral therapy
CVD	Cardiovascular disease
DHIS	District Health Information System
DR-TB	Drug-resistant tuberculosis
LMICS	Low- and middle-income countries
LTFU	Loss to follow up
MDR-TB	Multidrug-resistant tuberculosis
NCDs	Non-communicable diseases
Pre-XDR	Pre-extensively drug-resistant tuberculosis
RR-TB	Rifampicin-resistant tuberculosis
TB	Tuberculosis
WHO	World Health Organization
XDR-TB	Extensively-drug resistant tuberculosis
